# Molecular Characterization and Assessment of Insect Resistance of Transgenic Maize ZDRF-8

**DOI:** 10.3390/plants14060901

**Published:** 2025-03-13

**Authors:** Chengqi Zhu, Liang Qi, Yifan Yu, Xianwen Zhang, Jifeng Ying, Yuxuan Ye, Zhicheng Shen

**Affiliations:** 1 Institute of Insect Sciences, Zhejiang University, Hangzhou 310058, China; 12216133@zju.edu.cn (C.Z.); 22316092@zju.edu.cn (L.Q.); 22316239@zju.edu.cn (Y.Y.); 2 Institute of Virology and Biotechnology, Zhejiang Academy of Agricultural Sciences, Hangzhou 310021, China; bestzxw@163.com; 3 Hangzhou LeadGene Biotech Co., Ltd., Hangzhou 310018, China; yingjifeng@caas.cn; 4 The Rural Development Academy, Zhejiang University, Hangzhou 310058, China; yeyuxuan@zju.edu.cn

**Keywords:** transgenic maize, *Cry1Ab*, *Cry2Ab*, insect and herbicide resistance, ZDRF-8

## Abstract

ZDRF-8 is a transgenic maize event created via *Agrobacterium*-mediated transformation for insect resistance and glyphosate tolerance by expressing *Cry1Ab*, *Cry2Ab*, and *G10evo-epsps*. A Southern blot analysis suggested that it is a single-copy T-DNA insertion event. The flanking genomic sequences of the T-DNA insertion suggested that its T-DNA was inserted at the terminal region of the long arm of chromosome 7 without interrupting any known or predicted genes. Event-specific PCRs based on the flanking sequence were able to detect this event specifically. Laboratory bioassays and field trials of multiple generations demonstrated that ZDRF-8 is highly active against major corn pests in China, including Asian corn borers (ACB, *Ostrinia furnacalis*), cotton bollworms (CBW, *Helicoverpa armigera*), and oriental armyworm (OAW, *Mythimna separata*), and meanwhile confers glyphosate tolerance up to two times the recommended dose. The expression of the transgenes and the efficacy of insect resistance and glyphosate tolerance were stable over more than 10 generations. ZDRF-8 has been granted with a safety certificate in China, and its commercial release is expected in the coming years.

## 1. Introduction

Lepidoptera insects constitute predominant contributors to yield losses in maize cultivation, accounting for 6% of total production losses in North America and 13% in South America [1,2]. In China, lepidopteran-induced yield losses were estimated at 10% in spring maize and 20-30% in summer maize [3]. The first genetically modified (GM) insect-resistant maize was successfully developed and commercially released in the United States in 1996. The earliest commercialized maize, MON810, expresses the CryIA(b) protein, which has a toxic effect on Lepidoptera insects, especially the European corn borer. After the larvae of the European corn borer feed on the MON810 maize, the CryIA(b) protein will bind to the specific receptors on the epithelial cells of the insect’s midgut, damaging the cell structure, causing the insect’s intestine to perforate, stopping it from feeding, and ultimately leading to its death [4]. Since then, transgenic technology has emerged as the predominant method for controlling maize lepidopteran pests. Numerous transgenic maize varieties expressing diverse insecticidal proteins have been subsequently developed and commercialized. Initially adopted in USA, this innovation has been successfully implemented by major maize-producing nations like Brazil and Argentina, with subsequent global adoption expanding to more than five countries. This technological advancement is widely recognized as a seminal breakthrough in agricultural biotechnology [5].

The Cry protein has a highly specific toxic effect on insect larvae, which can cause lethal effects on the larvae of different insect species, but it has no such effect on humans or other mammals [6]. Generally, Bacillus thuringiensis secretes a series of insecticidal proteins, including toxins produced during the vegetative growth phase (such as secreted insecticidal protein, Sip; vegetative insecticidal proteins, Vip), parasporal crystalline δ-endotoxins produced during the stationary phase of vegetative growth (such as cytolytic toxin, Cyt, and crystal toxin, Cry), and β-exotoxins [7]. The first product based on the toxins of Bacillus thuringiensis (*Bt*) was commercialized in France in 1938 for the control of moths. In 1958, Bt products started to be commercially available in the United States [8]. The main application of Bt toxins is to control agricultural pests, and this pest control work is carried out through genetically modified plants (*Bt* plants). At present, a large number of Cry insecticidal toxin proteins have been discovered and applied to insect control. For example, proteins such as Cry1A, Cry2A, Cry3A, and Cry14A can be used to control insects of the order *Coleoptera*, while proteins like Cry1A, Cry2A, Cry4A, and Cry10A can be used to control insects of the order *Diptera*. Additionally, proteins including Cry3A, Cry5A, and Cry22A can be used to control insects of the order *Hymenoptera* [9].

Corn (*Zea mays* L.) is the biggest crop by acreage in China. With little doubt, transgenic insect resistance technology could be the most efficient and environment friendly method to control the lepidopteran insect pests in corn in China too. Therefore, it will be highly valuable to develop transgenic insect-resistant corn to control the lepidopteran pests, especially the Asian corn borer (ACB, *Ostrinia furnacalis*), cotton bollworm (CBW, *Helicoverpa armigera*), and oriental armyworm (OAW, *Mythimna separata*), which cause severe damage in China.

Cry1Ab is an effective *Bt* toxin against major lepidopteran pests on maize. Transgenic maize Bt11 and Mon810 expressing Cry1Ab have been commercially released and demonstrated great effectiveness in controlling the European corn borer (ECB, *Ostrinia nubilalis*) [10,11]. Cry1Ab was also found to be effective in controlling other lepidopteran species, such as corn earworm (CEW, *Helicoverpa zea*) and western bean cutworm (WBC, *Striacosta albicosta*) [2]. According to relevant reports, Cry1Ab has a very strong activity against the ACB and the OAW [12,13]. Cry2Ab is another Bt toxin that is also quite effective against ECB, CBW, OAW, and fall armyworm (FAW, *Spodoptera frugiperda*) [14]. Cry2Ab was utilized to molecular stacking with Cry1.105 in transgenic corn event Mon89034 [15].

Resistance development by insect pests to *Bt* corn is a major threat for the long-term utilization of *Bt* corn. The stacking of two *Bt* toxins without cross resistance is regarded as an effective strategy to mitigate the development of resistance by insect pests to *Bt* toxins [16]. Considering that Cry1Ab and Cry2Ab are both active to the three most important corn lepidopteran pests (ACB, CBW, and OAW) in China. we decided to develop a transgenic event that simultaneously expresses these two *Bt* toxins. An event named GAB-3 was selected, and its resistance to insect pest was evaluated primarily. In this study, we report the molecular characterization and evaluation of insect resistance and herbicide tolerance over multiple generations of GAB-3. This event was granted with a safety certificate in China under the name of ZDRF-8.

## 2. Results

### 2.1. Creation of Transgenic Plants Expressing Both Cry1Ab and Cry2Ab

**Figure 1:**
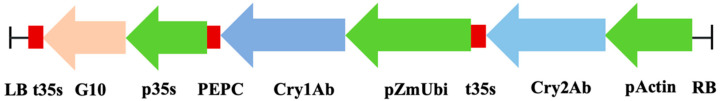
Schematic diagram of the T-DNA harboring *Cry1Ab*, *Cry2Ab* and *G10evo-epsps* expression cassettes. LB, left border of T-DNA; t35s, CaMV 35s terminator; G10, *G10evo-epsps* gene; p35s, CaMV 35s-Actin-intron promoter; PEPC, Mazie PEPC terminator; *Cry1Ab*, insecticidal *Cry1Ab* gene; pZmUbi, maize ubiquitin promoter; *Cry2Ab*, insecticidal *Cry2Ab* gene; pActin, rice Actin-1 promoter; RB, right border of T-DNA.

To generate transgenic maize expressing two insecticidal proteins (Cry1Ab and Cry2Ab), we constructed a T-DNA containing three expression cassettes for *Cry1Ab*, *Cry2Ab*, and *G10evo-epsps* (Figure 1). The rice actin promoter, maize Ubiquitin-1 promoter, and CaMV 35S promoter were employed to drive the expression of *Cry2Ab*, *Cry1Ab*, and *G10evo-epsps*, respectively. *G10evo-epsps* was used as a selection marker during transformation, with glyphosate used as the selective agent.

### 2.2. Molecular Characterization of Transgenic Event ZDRF

#### 2.2.1. Determination of Copy Number of Transgenes

**Figure 2:**
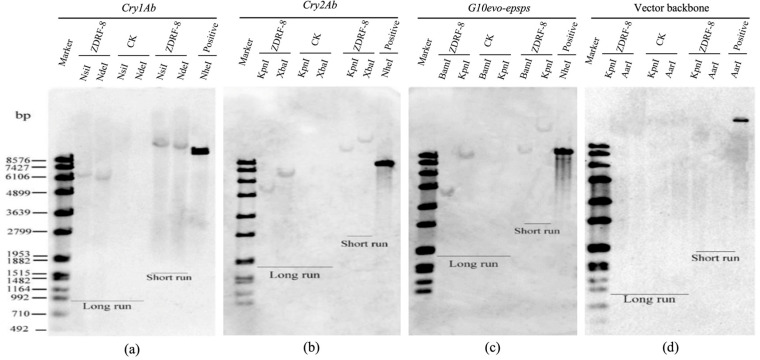
Southern blot analysis of the transgenes in ZDRF-8. Positive control: The corresponding exogenous protein products. CK: The corresponding non-transgenic maize Reuifeng-1. (**a**) The genomic DNA probed by *Cry1Ab* after digestion with *NdeI* and *NsiI*, respectively. (**b**) The genomic DNA probed by *Cry2Ab* after digestion with *KpnI* and *XbaI*, respectively. (**c**) The genomic DNA probed by *Cry2Ab* after digestion with *KpnI* and *BamHI*, respectively. (**d**) The genomic DNA probed by backbone of the transformation vector after digestion with *KpnI* and *AarI*, respectively. The transformation vector digested with *NdeI* served as the positive control. Long run sample: the sample was added normally; short run sample: the sample was loaded 6 h later.

To determine transgene copy numbers, a Southern blot analysis was performed using probes specific to *Cry1Ab*, *Cry2Ab*, and *G10evo-epsps*. Genomic DNA from ZDRF-8 digested with *NdeI* or *NsiI* exhibited single hybridization bands when probed with *Cry1Ab* (Figure 2a). Consistent results were obtained for *Cry2Ab* (Figure 2b) and *G10evo-epsps* (Figure 2c), where restriction enzyme-digested DNA showed singular hybridization signals regardless of enzyme selection. These Southern analyses conclusively demonstrated the single-copy integration of all transgene in the maize genome, which was further corroborated by quantitative PCR (qPCR) results.

To assess potential vector backbone transfer, specific probes targeting non T-DNA regions of the binary vector were hybridized with ZDRF-8 genomic DNA. The absence of detectable signals (Figure 2d) confirmed the successful elimination of backbone sequence transfer during the transformation process.

#### 2.2.2. Determination of Integration Site of T-DNA in Corn Genome

The flanking sequences of T-DNA in ZDRF-8 were successfully obtained through high-efficiency thermal asymmetric interlaced PCR (hiTAIL-PCR). A comparative analysis with the B73 maize reference genome revealed the T-DNA insertion site located at the distal end of chromosome 7. A genomic characterization identified a 17 bp sequence deletion at the insertion locus. Crucially, this insertion site resides in intergenic regions, with no predicted open reading frames (ORFs) or annotated functional elements detected within 10 kb upstream or downstream through a BLAST analysis (https://blast.ncbi.nlm.nih.gov/Blast.cgi, accessed on 9 March 2025). This genomic positioning suggests a minimal likelihood of disrupting endogenous gene functions or generating novel chimeric/fusion proteins.

The complete transgenic insert measured 11,064 bp in length, exhibiting characteristic border modifications: 11 bp and 49 bp deletions at the left and right T-DNA borders, respectively. These truncations align with typical *Agrobacterium*-mediated transformation patterns. The remaining sequence of the T-DNA in ZDRF-8 was completely identical to the T-DNA sequence in the transformation vector.

### 2.3. Analysis of Genetic Stability and Protein Expression Stability

**Figure 3:**
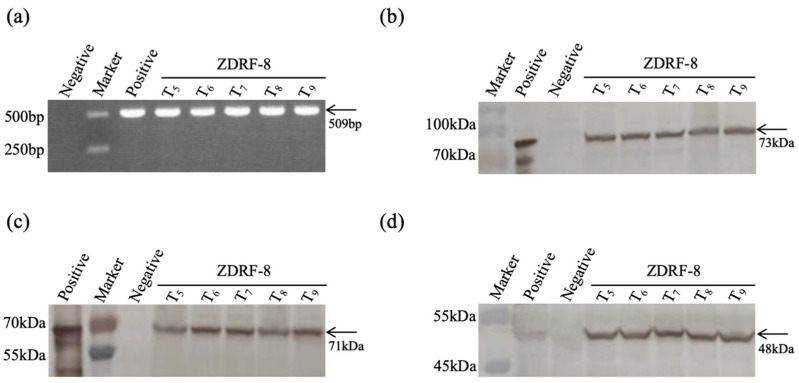
The genetic stability and protein expression stability of the transgenes in ZDRF-8. Positive control: The corresponding exogenous protein products. Negative control: The corresponding non-transgenic maize Reuifeng-1. (**a**) Specific PCR detection of ZDRF-8 across multiple generations, in which Ruifeng-1 genome sample served as the negative control and T_1_ ZDRF-8 genome sample served as the positive control. (**b**–**d**) The expression of *Cry1Ab* (**b**), *Cry2Ab* (**c**), and *G10evo-epsps* (**d**) proteins detected by Western blot analysis, in which the recombinant *E. coli* of *Cry1Ab*, *Cry2Ab*, or *G10evo-epsps* proteins were loaded as the positive controls and the non-transgenic Ruifeng-1 was loaded as the negative control.

To study the integration stability of transgenes across various generations of ZDRF-8, event-specific PCR analysis was performed on five generations (5th to 9th) of the transgenic maize ZDRF-8. The results showed that the transgenes were stably integrated over the five generations (5th to 9th) (Figure 3a). Furthermore, the expression of *Cry1Ab*, *Cry2Ab*, and *G10evo-epsps* proteins in ZDRF-8 were evaluated using a Western blot analysis, and the results suggested that the expression levels of these three proteins in ZDRF-8 were largely consistent over five generations (Figure 3b–d).

### 2.4. The Results of ELISA Detection for ZDRF

The results indicated that the expression levels of Cry1Ab, Cry2Ab, and G10evo-EPSPS could be detected in various tissues of transgenic insect-resistant maize GAB-3 at different growth stages. Cry1Ab had the highest expression level in leaves (VT), at 41.24 ± 4.05 μg/g fw, and the lowest in seeds (R6), at 1.96 ± 1.10 μg/g fw. Cry2Ab had the highest expression level in leaves (VT), at 4.96 ± 2.24 μg/g fw, and the lowest in roots (R6), at 1.34 ± 0.88 μg/g fw. G10evo-EPSPS had the highest expression level in leaves (VT), at 8.43 ± 2.26 μg/g fw, and the lowest in roots (R6), at 1.88 ± 0.86 μg/g fw. The edible part of transgenic insect-resistant maize GAB-3 is the maize grain, while the feed parts include leaves, stems, and grains. Cry1Ab expression in leaves ranged from 13.05 to 48.34 μg/g fw, in stems from 6.67 to 29.09 μg/g fw, and in grains from 0.68 to 4.33 μg/g fw. Cry2Ab expression in leaves ranged from 1.13 to 7.07 μg/g fw, in stems from 0.23 to 6.35 μg/g fw, and in grains from 0.85 to 4.22 μg/g fw. G10evo-EPSPS expression in leaves ranged from 2.56 to 11.35 μg/g fw, in stems from 0.76 to 6.82 μg/g fw, and in grains from 0.56 to 4.06 μg/g fw.

### 2.5. Evaluation of Insect Resistance of Transgenic Maize ZDRF-8

The *Bt* maize line ZDRF-8 expressing Cry1Ab, Cry2Ab, and G10evo-epsps proteins demonstrated robust tissues protection against the first-stage larvae of three lepidopteran pests. When fed fresh tissue samples (V5 leaves, R1 silks, and R2 kernels) from ZDRF-8 maize under controlled in vitro conditions, ACB, CBW, and OAW exhibited 100% mortality (± 0.00%) within three days. This mortality rate was significantly higher (*p* < 0.05, *n* = 30; Table 1) than that in non-*Bt* maize Ruifeng-1 controls, which showed mortality rates ranging from 20.38% to 49.50% across different tissues. The consistent efficacy across three phenological stages confirms stage-independent insect resistance in this transgenic event.

**Figure 4:**
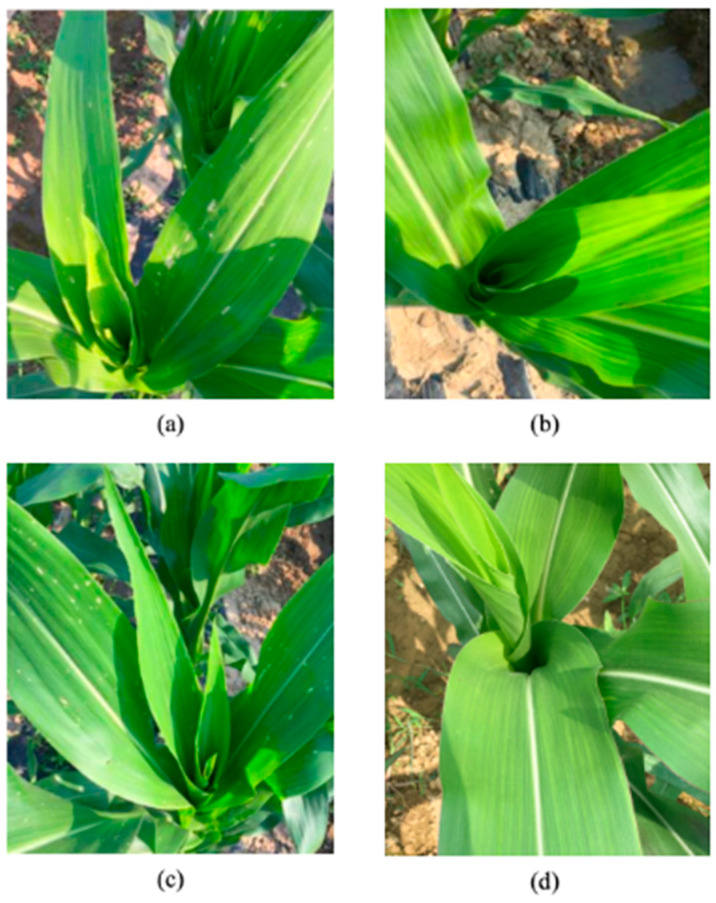
The damage situation of maize two weeks after insect inoculation at the early stage of the whorl leaf in the field experiment. (**a**) The damage situation of the non-transgenic maize Reifeng-1 two weeks after inoculation with OAW. (**b**) The damage situation of the transgenic maize event ZDRF-8 two weeks after inoculation with OAW. (**c**) The damage situation of the non-transgenic maize Reifeng-1 two weeks after inoculation with ACB. (**d**) The damage situation of the transgenic maize event ZDRF-8 two weeks after inoculation with ACB.

Following field inoculation with neonate larvae of CBW, *Bt* maize ZDRF-8 exhibited complete foliar protection with no visible feeding damage, whereas non-*Bt* maize Ruifeng-1 controls displayed characteristic injury patterns including circular perforations and irregular leaf margin consumption. Similar resistance patterns were observed against the inoculation of ACB and OAW. A quantitative assessment revealed significantly higher leaf damage scores in non-*Bt* plants (4.39 ± 0.42) compared to *Bt* counterparts (0.42 ± 0.17) (*p* < 0.05, *n* = 60; Table 2). Figure 4 shows the damage situation of maize two weeks after inoculation with OAW and ACB at the early stage of the whorl leaf.

### 2.6. Glyphosate Tolerance Analysis

**Figure 5:**
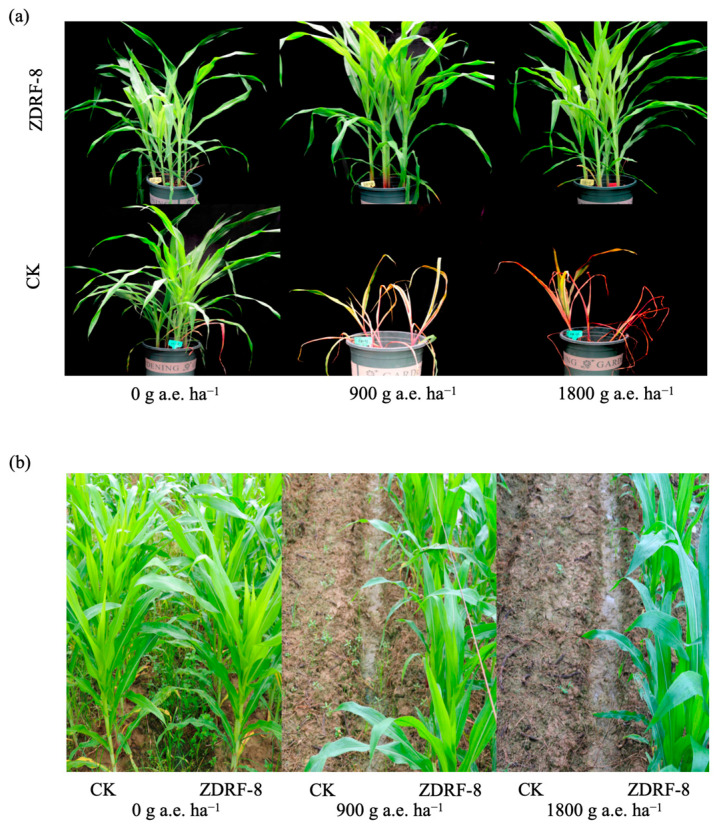
Glyphosate tolerance analysis in ZDRF-8. CK: The corresponding non-transgenic maize Reuifeng-1. (**a**,**b**) Tolerance of the transgenic maize ZDRF-8 and Ruifeng-1 to 0 g a.e. ha^−1^, 900 g a.e. ha^−1^, and 1800 g a.e. ha^−1^ of glyphosate in the greenhouse (**a**) and in the field (**b**), which was recorded two weeks after the application of glyphosate.

To evaluate the herbicide tolerance of transgenic maize ZDRF-8 to glyphosate, both field trials and controlled-environment experiments were implemented. Following the foliar application of glyphosate at 900 g a.e. ha^−1^ and 1800 g a.e. ha^−1^, transgenic maize ZDRF-8 maintained normal growth parameters without observed phytotoxic symptoms after two weeks, comparable to water-treated control plants (Figure 5a,b). In stark contrast, non-transgenic maize Ruifeng-1 exhibited complete mortality within the same observation period at both glyphosate concentrations, demonstrating absolute susceptibility to the herbicide (Figure 5a,b). These findings collectively indicate that ZDRF-8 manifests superior glyphosate tolerance relative to conventional maize varieties, sustaining unimpaired development even at the elevated glyphosate dosage of 1800 g a.e. ha^−1^.

## 3. Materials and Methods

### 3.1. Vector Construction and Maize Transformation

The full-length coding sequences of the *Cry1Ab*, *Cry2Ab*, and *G10evo-epsps* genes were optimized based on the codon bias of maize and synthesized by Sangon Biotech Co., Ltd. (Shanghai, China). The *Hygromycin* gene of the T-DNA vector pCAMBIA1300 was replaced with the glyphosate tolerance gene *G10evo-epsps*. This modified pCAMBIA1300 vector was further inserted by two expression cassettes of *Cry1Ab* and *Cry2Ab*, respectively. The *Cry1Ab* cassette consists of the maize ubiquitin promoter (pZmUbi), the *Cry1Ab* coding sequence, and the maize PEPC terminator (PEPC) from the 5′ to 3′ end. The *Cry2Ab* cassette consists of the rice actin-1 promoter (pActin), the *Cry2Ab* coding sequence, and the 35S gene terminator of CaMV. The transformation construct was named as pZDRF, and its T-DNA region was illustrated in Figure 1. The method of maize transformation generally follows Ishida’s Agrobacterium-mediated transgenic maize transformation method [17]. However, in this study, we used 2 mM of glyphosate to screen for positive plants.

### 3.2. Trait Integration

The T_0_ transgenic events were first screened by bioassays with ACB and then by qPCR to select events with only single copy of T-DNA insertion. The selected events were backcrossed into elite inbred line Zheng58. After performing backcross and then self-pollination three times, the transgenic homozygous plants of Zheng58 genetic background were obtained. The segregated non-transgenic plants were also saved as the non-transgenic control, and named as Ruifeng-1.

### 3.3. Southern Blot Analysis

Genomic DNA was isolated from leaf tissues of ZDRF-8 and non-transgenic plants Ruifeng-1 using the cetyltrimethylammonium bromide (CTAB) method [18], and was then digested overnight with restriction enzymes. After the digestion, genomic DNA was separated in 7 g/L agarose gel at 30 V over 8 h, and transferred onto a Hybond N^+^ membrane (Amersham, UK). Hybridization probes, specific to *Cry1Ab*, *Cry2Ab*, *G10evo-epsps* genes and vector backbone, respectively, were synthesized using the PCR DIG Probe Synthesis Kit (Roche, Basel, Switzerland). The probes were hybridized to DNA on blots on the membrane in the hybridization solution. After washing the unhybridized probe, the blots were visualized in a Gel Logic 2200 imaging system (Kodak, Rochester, NY, USA) through chemiluminescence. To have a better resolution for both large small size bands, the ZDRF-8 samples digested with restriction enzymes were loaded for two time points for a long run and a short run. The short run could prevent the loss of smaller fragment bands, and the long run could achieve higher band resolution of large size bands.

### 3.4. Determination of Insertion Sites by hiTAIL-PCR

Based on the sequences of the ends of the T-DNA, nested primers (LB-SPI, LB-SP2a, LB-SPIII, RB-0b, RB-1b, and RB-2b), random primers (LAD1/2/3/4), and the tag sequence AC1 were designed for high-efficiency TAIL-PCR (hiTAIL-PCR) (primers are listed in Table 3). Following the method of hiTAIL-PCR described by Liu [19]. The specific reaction systems and reaction steps are shown in Table 4, Table 5, Table 6 and Table 7. The actual maize genomic flanking sequences to the T-DNA were determined. The flanking maize genomic sequences were subjected to a BLAST analysis to the B73 RefGen_v2 (MGSC) database, and the insertion site of the T-DNA on the genome was determined.

### 3.5. Event-Specific PCR for Transgenic Plant Identification

The genomic DNA samples were isolated from leaf tissues of ZDRF-8 (total of 5 generations, from 5th to 9th) and the non-transgenic recipient plants Ruifeng-1 by the cetyltrimethylammonium bromide method (CTAB) [18], and were used as templates for event-specific PCR to identify transgenic plants. The genomic DNA of Ruifeng-1 was served as a negative control. The event-specific PCR primers were designed based on the flanking genomic sequences and their nearby T-DNA sequences (listed in Table 8). The PCR was conducted using the following parameters: 94 °C for 2 min; 30 cycles of 94 °C for 30 s, 55 °C for 30 s, and 72 °C for 2 min.

### 3.6. Western Blot Analysis

Fresh leaves of 5 generations of ZDRF-8 and non-transgenic control maize Ruifeng-1 were sampled for Western blot analysis. The sample leaves were added with steel balls and then subjected to sample crusher machine (45 Hz, 45 s) to grind into powder. The fine powders of the samples were then suspended into phosphate-buffered saline (PBS). Add 10 μL of 5× protein loading buffer (containing β-mercaptoethanol) and mix well. Boil in boiling water for 10 min and centrifuge at 12,000 rpm for 5 min. Take an appropriate amount of supernatant for electrophoresis. The protein samples were used to perform a Western blot analysis, following the protocol described by Liu [20]. The polyclonal antibodies against insecticidal proteins Cry1Ab and Cry2Ab and the herbicide protein G10evo-epsps were diluted 250 times for incubation in a Western blot analysis, and they were prepared by the Zhejiang Chinese Medical University after four immunizations of New Zealand white rabbits. The goat anti-rabbit horseradish peroxidase (HRP) conjugate was used as secondary antibody, and 3,3′-diaminobenzidine (DAB) was used for the final chromogenic reaction. The expression of Cry1Ab, Cry2Ab, and G10evo-epsps in ZDRF-8 was detected by Western blot analysis.

### 3.7. Bioassay of ZDRF Resistance to Three Species of Lepidoptera Pests

Tissue samples of various types—V5 leaves, R1 silks, and R2 kernels—were sampled from the ZDRF-8 maize and non-Bt maize Ruifeng-1 from a field trial and were taken immediately to the laboratory for bioassays against neonates larvae of ACB, CBW, and OAW. Maize tissue samples were placed in 24-well culture plates. Six neonates (0–24 h) were transferred to each well with a fine brush, and the plates were sealed with Para-film to prevent the larvae from escaping and to keep them moisturized. Each treatment was replicated five times for each maize variety, and total of 30 larvae were tested for each treatment. All plates were placed at a temperature of 28 °C and a relative humidity of 80%, with a photoperiod of 16 h/8 h (L/D). Mortality rates were calculated after 3 days. For better evaluation of the insecticidal effects, mortality rates were corrected by employing Abbott’s formula, which accounted for the natural mortality recorded in the control group [21]. The larvae in the assays that exhibited no response even upon gentle brushing were deemed deceased.

The experiment involved a randomized complete block design for both the ZDRF-8 corn and conventional maize Ruifeng-1. After ZDRF-8 maize and non-Bt maize Ruifeng-1 were sown, each block (5 m × 6 m) was completely covered with 80-mesh gauze to isolate against interference from other lepidopteran larvae. The rows were spaced 60 cm apart, and the plants within each row were spaced 25 cm apart. Isolation belts were set up within the experimental blocks to prevent cross-contamination. Conventional cultivation methods and management practices were employed, and no insecticides were applied throughout the entire growth period. When maize plants reached the V5 stage, the second or sometimes third plants of a single spot were deselected due to multiple seeding, and 60 maize plants were retained in each block. Subsequently, 30 first-instar larvae were manually inoculated onto the whorl of each maize plant using a soft-bristled brush. After the inoculation, the leaf damage score was surveyed every two days. The leaf damage score was determined by following the standards outlined in the Ministry of Chinese Agriculture Bulletin [22]. The tests were repeated three times.

Using the SPSS 29.0 (IBM, Armonk, NY, USA) system, a variance analysis was performed on all data. The Duncan’s Test was employed for multiple comparisons of field insect resistance data, while a one-way ANOVA was used to analyze indoor insect resistance data.

### 3.8. Evaluation of Glyphosate Tolerance of ZDRF

In the field trial for glyphosate tolerance, the transgenic maize ZDRF-8 and non-GMO maize Ruifeng-1 were planted using a completely randomized block design (plots of rows spaced 0.6 m apart covered an area of 24 m^2^) with three replicates. The recommended dose of glyphosate was 100-fold dilution of glyphosate isopropylamine salt (41%, Roundup, Monsanto, Saint Louis, MO, USA), and three concentrations (0 g a.e. ha^−1^, 900 g a.e. ha^−1^, 1800 g a.e. ha^−1^) were applied to maize at the 3–4 leaf stage. Herbicide resistance assessments of ZDRF-8 and Ruifeng-1 were also conducted in the laboratory, in which the 3–4 leaf stage maize was pot-planted with three replicates in a greenhouse. The herbicide resistance and weed control efficacy were evaluated 14 days after the application of glyphosate in both field and laboratory.

### 3.9. ELISA Detection of ZDRF

The expression levels of Cry1Ab, Cry2Ab, and G10evo-EPSPS in transgenic maize ZDRF-8 were measured using the enzyme-linked immunosorbent assay (ELISA). The kits for measuring Cry1Ab and Cry2Ab were purchased from EnviroLogix (Portland, ME, USA) (Catalog Numbers AP-003-CRBS and AP-005-CT, respectively); the ELISA kit for measuring G10evo-EPSPS was developed and prepared by Youlong Biotech (Shanghai, China) The specific methods and procedures for the measurements followed the protocols provided with the kits. The transgenic maize ZDRF-8 samples were sourced from a transgenic trial base of Zhejiang University Agricultural Experiment Station, planted in Xinfeng Village, Sian Town, Changxing County, Zhejiang Province. The trial used a randomized block design with four replicates. Test samples were collected from five different plants at the trial site, and the average and standard deviation were calculated after measurement. The collected plant tissues mainly included leaves (V3, V6, V12, VT, and R3), roots (V3, V6, V12, VT, and R3), stems (V3, V6, V12, VT, and R3), pollen (VT), styles (R1), and grains (R6). All collected samples were thoroughly ground under liquid nitrogen and stored as powdered samples at −80 °C before measurement. The protein expression levels in each plant tissue were measured based on micrograms of protein per gram of fresh weight (μg/g fw). By calculating the moisture content in each tissue, the dry weight conversion factor was obtained, and the measured exogenous protein content was converted to micrograms of protein per gram of dry weight (μg/g dw).

## 4. Discussions

The T_0_ transgenic plants generated by *Agrobacterium*-mediated transformation were initially screened based on three key parameters: insect resistance efficacy, expression levels of insecticidal proteins, and T-DNA insertion copy numbers. Events exhibiting both strong insect resistance and a single T-DNA insertion copy were prioritized for comprehensive characterization. This included the identification of flanking genomic sequences. Ultimately, the ZDRF-8 event was selected as the lead candidate based on two decisive factors: (1) demonstration of superior insecticidal activity compared to other events, and (2) confirmation through a genomic analysis that the T-DNA insertion did not disrupt any known or predicted gene sequences in the host genome.

Cry1Ab and Cry2Ab are widely commercialized *Bt* toxins in maize for their high activity to major corn lepidopteran insect pests. However, the development of resistance in pests to Cry1Ab has significantly compromised its field effectiveness [23]. Field-evolved resistance to Cry2Ab was also first reported in Australian *Helicoverpa punctigera* populations (2002), followed by subsequent confirmation in Chinese *H. punctigera* populations (2004). Practical resistance to Cry2Ab has been confirmed in Indian *Pectinophora gossypiella* populations infesting *Bt* cotton fields [24]. These documented resistance cases highlight the urgent need for the deployment of pest resistance management strategies, such as toxin pyramiding.

Theoretical model studies indicate that “pyramided” plants expressing two distinct *Bt* toxin genes can delay pest resistance more effectively than those employing a single toxin [25]. There are reports indicating that plants containing two different Bt toxin genes, Cry1Ac and Cry1C (“pyramided” plants), compared with single-toxin plants used sequentially or in a mosaic planting pattern, can significantly delay the time when the diamondback moth (*Plutella xylostella*) develops resistance to the pyramided plants with two genes after 24 generations of screening [26]. This strategy also broadens the spectrum of pest insect and enhances the efficacy [27]. Therefore, we used both Cry1Ab and Cry2Ab to develop the transgenic maize ZDRF-8.

Bacillus thuringiensis is an invertebrate pathogen that can infect a variety of insects, mainly mediated by the activity of crystal Cry toxins. These toxins are produced simultaneously during the process of spore formation. The host range of a specific Bacillus thuringiensis strain is highly specific and largely depends on the specific Cry toxins it produces [28].

Many proteins of the Cry family have been used to cultivate transgenic insect-resistant maize. The transgenic maize events OE1 and OE3 expressing the Cry1Ah-1 protein are expressed in different tissues during the six-leaf stage, heading stage, and grain-filling stage, which can ensure that the maize is protected from corn borers throughout the entire growth cycle. Indoors, the mortality rate of corn borers that feed on the leaves of OE1 and OE3 maize reaches 100% after 3 days. In the field, they also have a strong insecticidal activity against corn borers, reaching a high resistance level [29]. The transgenic maize events CVC-1 and CVC-2 expressing the exogenous proteins of Cry2Ab and Vip3A showed strong insecticidal toxicity against *Mythimna separata*, *H. punctigera*, and FAW. Six days after the occurrence of the insect infestation, the mortality rates of these insects exceeded 96%, 100%, and 100%, respectively [30].

Numerous experiments and practices have shown that Cry proteins can effectively control pests such as Lepidoptera or Coleoptera. Transgenic Bt crops can bring benefits to the environment and the economy by reducing the environmental pollution caused by chemical pesticides and improving the quality and yield of crops. However, due to concerns such as the evolution of pest resistance and other existing issues, the cultivation of Bt crops still remains controversial [31]. To evaluate the safety of insect-resistant transgenic crops for non-target organisms, it is not feasible to test every arthropod species in the field due to their extreme diversity [32]. Therefore, it is necessary to select representative species for testing. In the following research, the safety assessment of the transgenic maize event ZDRF-8 for non-target organisms will continue.

The molecular characterization of ZDRF-8 demonstrated the single-copy T-DNA integration of three transgenes (*Cry1Ab*, *Cry2Ab*, and *G10evo-epsps*) into the maize genome. A border sequence analysis revealed that the T-DNA was not inserted into a known or predicated gene, and thus, it is unlikely to exert unintended biological effects on maize [33]. Our analysis of the stability of transgene expression and insect resistance across multiple generations suggested that the T-DNA insertion site of ZDRF-8 is favorable.

Our previous study demonstrated that transgenic maize ZDRF-8 exhibits potent insecticidal activity against four major lepidopteran corn pests: OAW, ACB, CBW, and FAW [34]. Data from this study further showed that the strong resistance of ZDRF-8 to lepidopteran insects was stable for multiple generations, suggesting that there was no significant gene silencing of the transgenes of this event. The combination of Cry1Ab and Cry2Ab not only provided high resistance to major lepidopteran pests in corn but also effectively expanded the insecticidal spectrum to include earworm and cutworm [30].

## Figures and Tables

**Table 1:** The survival rates of newly hatched larvae of three kinds of pests after they fed on various tissues of ZDRF-8 and its non-transgenic control maize for three days.

Pests	Maize Tissue	Survival Rate of Larvae/%	
		ZDRF-8	Reifeng-1
ACB	Leaf	100.00 ± 0.00 a	20.38 ± 3.57 d
	Silk	100.00 ± 0.00 a	32.37 ± 5.27 c
	Kernel	100.00 ± 0.00 a	49.50 ± 4.60 a
CBW	Leaf	100.00 ± 0.00 a	30.08 ± 4.75 c
	Silk	100.00 ± 0.00 a	27.34 ± 5.18 c
	Kernel	100.00 ± 0.00 a	41.30 ± 4.85 b
OAW	Leaf	100.00 ± 0.00 a	40.00 ± 1.80 b
	Silk	100.00 ± 0.00 a	33.40 ± 5.15 c
	Kernel	100.00 ± 0.00 a	42.80 ± 2.96 b

The data in the table are in the format of mean ± standard deviation. The ANOVA analysis method was adopted. The lowercase English letters in the same column indicate significant differences after the DUCAN post hoc multiple comparison (*p* < 0.05, *n* = 30).

**Table 2:** The survival field resistance of ZDRF-8 and its non-transgenic control maize to ACB, CBW, and OAW during the whorl leaf stage after two weeks.

Pest	Maize Material	Leaf Damage Score	Resistant
ACB	ZDRF-8	1.07 ± 0.12 b	High resistance
	Reifeng-1	4.39 ± 0.42 a	Resistance
CBW	ZDRF-8	0.42 ± 0.17 c	High resistance
	Reifeng-1	3.72 ± 0.31 a	Resistance
OAW	ZDRF-8	0.85 ± 0.08 b	High resistance
	Reifeng-1	3.68 ± 0.12 a	Resistance

The data in the table are in the format of mean ± standard deviation. The ANOVA analysis method was adopted. The lowercase English letters in the same column indicate significant differences after the DUCAN post hoc multiple comparison (*p* < 0.05, *n* = 60).

**Table 3:** hiTAIL-PCR primer sequence.

Primer	Primer Sequences (5′-3′)
LB-SPI	TTTCTCCATAATAATGTGTGAGTAGTTCCC
LB-SP2a	ACGATGGACTCCAGTCCGGCCCTCATGTGTTGAGCATATAAGAAACCCTTAG
LB-SPIII	CTAAAACCAAAATCCAGTACTAAAATCC
RB-0b	CGTGACTGGGAAAACCCTGGCGTT
RB-1b	ACGATGGACTCCAGTCCGGCCCAACTTAATCGCCTTGCAGCACATC
RB-2b	GAAGAGGCCCGCACCGATCGCCCTT
LAD1	ACGATGGACTCCAGAGCGGCCGCVNVNNNGGAA
LAD2	ACGATGGACTCCAGAGCGGCCGCBNBNNNGGTT
LAD3	ACGATGGACTCCAGAGCGGCCGCVVNVNNNCCAA
LAD4	ACGATGGACTCCAGAGCGGCCGCBDNBNNNCGGT
AC1	ACGATGGACTCCAGAG

**Table 4:** hiTAIL-PCR pre-amplification.

Component	Volume (µL)
PrimeSTAR^®^ HS DNA polymerase	0.25
2×GC Buffer	12.5
Prime Star dNTP	2
LB-SPI/RB-0b (10 µmol/µL)	0.5
LAD mix (LAD1-1/2/3/4) (10 µmol/µL)	2
Genomic DNA	1
ddH_2_O	6.75

**Table 5:** hiTAIL-PCR primary amplification.

Component	Volume (µL)
PrimeSTAR^®^ HS DNA polymerase	0.25
2×GC Buffer	12.5
Prime Star dNTP	2
LB-SPII2a/RB-1b (10 µmol/µL)	0.5
AC1 (10 µmol/µL)	0.5
Template (first round product diluted 100 times)	1
ddH_2_O	8.25

**Table 6:** hiTAIL-PCR secondary amplification.

Component	Volume (µL)
PrimeSTAR^®^ HS DNA polymerase	0.5
2×GC Buffer	25
Prime Star dNTP	4
LB-SPIII/RB-2b (10 µmol/µL)	1
AC1 (10 µmol/µL)	1
Template (second round product diluted 20 times)	2
ddH_2_O	16.5

**Table 7:** hiTAIL-PCR reaction steps.

Pre-Amplification			Primary Amplification		Secondary Amplification	
Step	Temperature (°C)	Time	Temperature (°C)	Time	Temperature (°C)	Time
1	98	1:00	98	0:15	98	0:15
2	98	1:00	65	0:30	65	0:30
3	98	0:15	Go to step 1	1 time	72	1:30
4	60	0:30	72	1:30	98	0:15
5	72	1:30	98	0:15	68	0:30
6	Go to step 3	10 times	68	0:30	72	1:30
7	98	0:15	72	1:30	98	0:15
8	25	2:00	98	0:15	50	0:30
9	Ramping to 72	0.5 **°C**/S	68	0:30	72	1:30
10	72	3:00	72	1:30	Go to step 1	6 times
11	98	0:15	98	0:15	72	3:00
12	58	0:30	68	0:30	16	END
13	72	1:30	72	1:30		
14	Go to step 11	25 times	98	0:15		
15	72	5:00	50	0:30		
16	16	END	72	1:30		
17			Go to step 4	13 times		
18			72	3:00		
19			16	END		

**Table 8:** Transformation-specific PCR primer sequence.

Primer	Primer Sequences (5′-3′)
ZDRF8LB-F	CGTCCGCAATGTGTTATTAAGTTGTCTAAG
ZDRF8LB-R	AGTGCCAATACATACGCAACTGTTGCAG
ZDRF8RB -F	GCTGATCTGCTGCTCGTGCGAAAGTTAC
ZDRF8RB -R	GCAGCTTGAGCTTGGATCAGATTGTC

## Data Availability

Data is contained within the article.
